# Health insurance subscription among women in reproductive age in Ghana: do socio-demographics matter?

**DOI:** 10.1186/s13561-016-0102-x

**Published:** 2016-06-21

**Authors:** Hubert Amu, Kwamena Sekyi Dickson

**Affiliations:** Department of Population and Health, University of Cape Coast, Cape Coast, Ghana

**Keywords:** Ghana, Health insurance, Subscription, Women, Socio-demographics, Out-of-pocket payments

## Abstract

**Background:**

Premised that health insurance schemes in Africa have only been introduced recently and continue evolving, various concerns have been raised regarding their effectiveness in improving utilisation of orthodox health care and the reduction of out-of-pocket expenditures for their population, particularly women.

**Objective:**

To examine the effects of socio-demographics on health insurance subscription among women in Ghana.

**Methods:**

The study draws on the 2014 Ghana Demographic and Health Survey. Bivariate descriptive analysis and binary logistic regression were used to analyse the data.

**Results:**

Wealth status, age, religion, birth parity, marriage and ecological zone were found to have significantly predicted health insurance subscription among women in reproductive age in Ghana. Urban dwellers, women who are nulliparous, those with no or low levels of education, African traditionalists and the poor were those who largely did not subscribe to the scheme.

**Conclusion:**

The findings underscore the need for the National Health Insurance Authority to carry out more education in association with the National Commission for Civic Education and the Information Services Department to recruit more urban dwellers, nulliparous women, those with no or low levels of education, African traditionalists and the poor unto the scheme.

## Background

A major issue that continues to be of principal prominence in most countries across the globe entails the capacity of their health financing structures to provide adequate financial risk safeguard to all of their population against the costs of health care as they strive to achieve universal health coverage [1, 2]. In developing countries, health care accessibility remains limited as a result of financial and socio-cultural challenges. Out-of-pocket payments are among the main factors which prevent majority of the people in these countries from accessing timely health care [3]. This sometimes results in circumstances whereby enormous financial hurdles come upon entire countries through higher spending on treatment of ailments [4].

Out-of-pocket payments constitute a strong barrier to the utilisation of health care services, as well as precluding adherence to long term treatment especially among the vulnerable and poor [5]. Out-of-pocket payments for health care at the point of service delivery have also been troubling for the economic disposition of the poor, therefore, causing serious challenges with regards to essential daily needs as their incomes are drained by health care spending [6]. In order to substitute the user fees approach to health care financing and hence ameliorating the exorbitant health care cost for the masses, all member States of the World Health Organisation (WHO) adopted a resolution which aimed at encouraging nations to develop health financing systems with the aim of providing universal coverage [7]. One major approach to adequate health financing for many of these countries in efforts to achieve universal health coverage has therefore become health insurance [8]. There has been considerable interest in exploring the capabilities of health insurance in Africa, a continent which is considered to have a strong propensity for risk sharing across populations and time [9]. A number of African countries (Rwanda, Nigeria, Tanzania, Kenya and Ghana) are thus currently experimenting with various health insurance options which comprise both private and public schemes [10–14].

Premised that the health insurance schemes in Africa have been introduced within the last four decades and continue evolving, various concerns have been raised regarding their effectiveness in the reduction of out-of-pocket expenditures for their populations and improving utilisation of orthodox health care; a system in which health care professionals treat diseases and symptoms using radiation, drugs or surgery [15]. Access to effective health insurance has been noted to affect households by leading to better health especially among women who constantly need maternal and child health services as well as mitigating the risk of health shocks and reducing out-of-pocket health expenses [16, 17]. It is therefore imperative to understand the various factors which affect the ability of people, particularly women in their reproductive age to subscribe to health insurance.

Even though Ghana’s National Health Insurance Scheme (NHIS) became operational in 2003 through the National Health Insurance Law (Act 650 of Parliament), the scheme had a legal framework in 2004 through the National Health Insurance Regulations (L.I. 1809) [18, 19]. Financing of the NHIS is done by a 2.5 % insurance levy as Valued Added Tax (VAT) on goods and services, 2.5 % deductions from pension contributions of workers in the formal sector with the Social Security and National Insurance Trust (SSNIT) and yearly premiums paid by adults (persons eighteen years of age and above) [20]. The scheme is also financed through monies allocated to the Health Insurance Fund (HIF) by the legislature in addition to grants, investments, donations, voluntary contributions and gifts [21]. SSNIT pensioners, persons who are seventy years and above, children under the age of eighteen years and pregnant women, however, constitute exemptions on the NHIS from payment of yearly premium [20].

The National Health Insurance Scheme covers about 95 % of the disease burden of Ghana. These comprise services provided for out-patient clients such as diagnostic testing and operations including repair of hernia; most services for in-patient clients which include care by specialists, majority of surgeries and accommodation at the wards of health facilities; treatments for oral health; services related to maternal care including caesarean sections; emergency care; and all drugs that are listed on the medicines list of the National Health Insurance Scheme [22].

The institution mandated by law to manage the National Health Insurance Scheme is the National Health Insurance Authority (NHIA). The NHIA, in efforts to improve the scheme’s performance in meeting the health needs of Ghanaians, has since its inception in 2003, taken a number of initiatives. These include the free maternal health care introduced in 2008, a health insurance claims processing center established in 2010, introduction of clinical audit in 2010, creation of a consolidated premium account in 2011 and introduction of the national health insurance call center in 2012 [23]. The Authority also introduced biometric identification cards in 2014 to improve client identification and efficient delivery of services [24].

The National Health Insurance Scheme was designed to be pro-poor [20]. In practice, however, most subscribers to the scheme are people in the upper wealth quintile, as the poor and vulnerable including women in their reproductive years are rather less likely to subscribe to the scheme. The present study therefore sought to examine the determinants of health insurance ownership among women of reproductive age in Ghana with data from the 2014 Ghana Demographic and Health Survey. Several studies have investigated the influence of health insurance on other issues including utilisation of maternal health care services and medical out-of-pocket expenses [13, 22, 25–29].

Much attention has, however, not been paid to the background factors which influence subscription to the scheme. Even though Kumi-Kyereme and Amo-Adjei [30] examined factors influencing health insurance subscription in Ghana using data from the 2008 Ghana Demographic and Health Survey (GDHS), the authors focused mainly on spatial location and household wealth as principal determinants. The present study, however, examines the effects of all background characteristics on health insurance subscription and also compares the results to the study conducted by Kumi-Kyereme and Amo-Adjei [30] to identify variations in the factors influencing subscription in the 2008 and 2014 GDHS.

## Methods

### Source of data

The study made use of data from the female data file of the 2014 GDHS. The GDHS is a nationwide survey which covers all ten regions and is conducted every five years. The survey is carried out by the Ghana Statistical Service and the Ghana Health Service with ICF International providing technical support for the survey through MEASURE DHS. The GDHS focuses on child and maternal health and is designed to provide adequate data to monitor the population and health situation in Ghana. The survey gathers data on various demographic and health issues including fertility, contraceptive use, child health, nutrition, malaria, HIV and AIDS, family planning, health insurance and maternal health; antenatal care, delivery care and post-natal care. In the 2014 version 9396 women between the ages 15 and 49 were interviewed from 12,831 households covering 427 clusters throughout the country. The survey had a response rate of 97 % [31]. Permission to use the data set was given by the MEASURE DHS following the assessment of a concept note.

### Analysis

The dependent variable employed for this study was ownership of NHIS. The dependent variable was coded 1 = “Yes” and 0 = “No” since it was dichotomous. A discrete choice model was employed to show how the independent variables correlated with the dependent variable. Specifically, the binary logistic regression was employed since it allows the predictions on a mixture of continuous and categorical variables, given that this technique is more appropriate for dichotomous variables. A key assumption underlying the binary logistic regression model is that the dependent variable should be dichotomous in nature and the data should not have any outlier. The formulae underpinning the model is given as;

Let Y be a dichotomous variable which is defined as

$$ Y=\left\{\begin{array}{c}\hfill 1\kern0.24em for\kern0.24em  subscribers\hfill \\ {}\hfill 0\kern0.24em for\kern0.24em non\mathit{\hbox{-}} subscribers\hfill \end{array}\right. $$

and $$ \mathrm{p} = \Pr \left(\mathrm{Y} = 1\Big|{\mathrm{X}}_1,\dots, {\mathrm{X}}_{\mathrm{k}}\right) $$.

$$ \mathrm{p}=\frac{1}{1+ \exp \left[-\left({\upbeta}_0+{\upbeta}_1{\mathrm{X}}_1+{\upbeta}_2{\mathrm{X}}_2+\dots +{\upbeta}_{\mathrm{k}}{\mathrm{X}}_{\mathrm{k}}\right)\right]} $$

and $$ \widehat{\mathrm{p}}=\frac{1}{1+ \exp \left[-\left({\widehat{\upbeta}}_0+{\widehat{\upbeta}}_1{\mathrm{X}}_1+{\widehat{\upbeta}}_2{\mathrm{X}}_2+\dots +{\widehat{\upbeta}}_{\mathrm{k}}{\mathrm{X}}_{\mathrm{k}}\right)\right]} $$

Note: With no predictors, $\widehat{\mathrm{p}}=\frac{{\displaystyle \sum_{\mathrm{i}=1}^{\mathrm{n}}{\mathrm{Y}}_{\mathrm{i}}}}{\mathrm{n}}=\overline{\mathrm{Y}}$

Nine independent variables were used for the study; Maternal age, marital status, educational level, residence, wealth quintile, ethnicity, occupation, parity (Birth order) and region of residence. Maternal age was categorised into, 15–19, 20–24, 25–29, 20–34, 35–39, 40–44, and 45–49. Marital status was recoded as single (never married, widowed, divorced, not living together), married and cohabitation (living together). Education level was classified into four categories: No education, primary education, secondary education and higher education.

Type of residence was coded as urban and rural while wealth quintile was categorised as poorest, poorer, middle, richer and richest. Ethnicity was recoded as Akan, Ga/Dangme, Ewe, Guan, Mole–Dagbani, Grusi, Gurma and Other. Occupation was captured as not working and working. Parity (birth order) was categorised as zero birth, one birth, two births, three births and four births or more. Region of residence was re – coded to capture the general ecological zones as follows: Northern, Upper West and Upper East regions were re - coded as the ‘Savannah zone’; the Brong – Ahafo, Ashanti and Eastern regions were designated as the ‘forest zone’; while the Western, Central, Greater Accra and Volta regions were coded as the ‘coastal zone’. Religion was captured as Catholic, Anglican, Methodist, Presbyterian, Pentecostal/charismatic, Other Christian, Islam, African Traditional/spiritual and other. Survey weights, which are typical of nationally representative studies, were factored into both inferential and descriptive analyses conducted. The weights helped to offset the challenges of under and over sampling usually associated with national surveys. All analyses were conducted with STATA, version 13.

## Results

Table 1 presents respondents who were registered under the NHIS, based on various socio-demographic characteristics which comprised residence, ecological zone, age, ethnicity, occupation, education, religion, wealth status, marital status, and parity. In terms of religion, it was observed that less than half of the women in both rural (47.5 %) and urban (47.3 %) settlements had health insurance. While majority of women in the forest zone (64.2 %) had insurance, less than 50 % of their counterparts in the coastal (37 %) and savannah (39.5 %) were NHIS subscribers. Less than half of the respondents in their late teens (42 %), early thirties (42.7 %), and late forties (41.2 %) also had insurance.

**Table 1:** Insurance coverage and background characteristics

Variable	Correlates	*N* = 9363	Proportion registered	*P* value
Residence	Urban	789	47.48	0.050*
	Rural	724	47.25	
Ecological Zone	Coastal zone	616	36.97	0.000*
	Forest zone	763	64.18	
	Savannah zone	133	39.46	
Age	15–19	250	41.99	0.000*
	20–24	303	49.58	
	25–29	247	50.01	
	30–34	179	42.75	
	35–39	209	52.66	
	40–44	200	52.66	
	45–49	125	41.21	
Ethnicity	Akan	928	52.80	0.000*
	Ga/dangme	78	29.55	
	Ewe	150	38.55	
	Guan	31	45.08	
	Mole-Dagbani	193	51.32	
	Grusi	35	54.34	
	Gurma	52	27.59	
	Mande	20	76.18	
	Other	27	44.10	
Occupation	Not working	330	45.01	0.36
	Working	1181	47.34	
Education	No education	240	38.56	0.000*
	Primary	284	43.09	
	Secondary	922	51.83	
	Higher	68	50.15	
Religion	Catholic	147	56.08	0.000*
	Anglican	17	59.77	
	Methodist	125	58.91	
	Presbyterian	99	54.59	
	Pentecostal/charismatic	679	47.21	
	Other Christian	212	41.84	
	Islam	182	48.63	
	African Traditional/spiritual	13	17.60	
	Other	0	0	
Wealth status	Poorest	195	40.16	0.001*
	Poorer	274	43.64	
	Middle	348	48.44	
	Richer	379	51.80	
	Richest	318	50.24	
Marital status	Single	721	45.24	0.007*
	Married	540	50.06	
	Cohabitation	253	48.31	
Parity	No birth	465	43.26	0.001*
	One birth	202	50.24	
	Two births	204	52.53	
	Three births	185	50.76	
	Four births or more	457	47.39	

Source: GDHS 2014

Proportion registered* mean the proportion of women who had registered under the National Health Insurance Scheme (NHIS)

It was observed that below 50 % of respondents who were Ga/Dangmes (29.6 %), Ewes (38.6 %), Gurmas (27.6 %), and other (44.1 %) actually owned health insurance. Respondents with no education also had the lowest insurance ownership (38.6 %). It was also realised that health insurance subscription was higher for those working than those who were not working at the time of the survey. While over 50 % of married women were having health insurance, less than half of those single and cohabiting were subscribed to the scheme. African Traditionalists were the least of the respondents who owned health insurance. It was only respondents in the ‘Richer’ and ‘Richest’ wealth categories that majority had health insurance. In terms of parity, women who had never given birth were the least to have health insurance. Overall, 47.4 % of the weighted sample was registered under the scheme. It was observed from the Chi-square tests conducted that in exception of occupation, all the other background characteristics had significant associations with health insurance ownership.

Table 2 presents results of the Binary logistic regression of NHIS subscription among the women surveyed. The results reveal significant effects of wealth status (richer, richest), age (45–49), ecological zone (forest zone, savannah), religion (Pentecostal/charismatic, other Christian, African traditionalist/spiritualist, no religion), ethnicity (Ga/Dangme), education, marital status (married), and parity on subscription. Women in the rural areas were more likely to own health insurance compared with their colleagues in urban areas. Those in the ‘richer’ wealth quintile were found to be 170 % more likely to register with the NHIS than those in the ‘poorest’ wealth quintile. Respondents in the forest (OR = 2.863, CI = 2.393–3.426) and savannah (OR = 2.167, CI = 1.580–2.972) zones also had higher probabilities of owning health insurance compared with women in the coastal zone. Regarding religion, it was observed that only Anglicans (OR = 1.231, CI = 0.515–2.944) and Methodists (OR = 1.010, CI = 0.692–1.475) were more likely to own health insurance than Catholics. Irrespective of the number of births, it was observed that respondents who had ever given birth were all at least 140 % more likely to own health insurance compared with those who had never given birth (Table 2).

**Table 2:** Binary logistic regression on ownership of health insurance

Independent variable	Odds ratio	95 % confidence interval
Residence		
Urban	Ref	Ref
Rural	1.163	0.950–1.423
Wealth status		
Poorest	Ref	Ref
Poorer	1.058	0.811–1.381
Middle	1.235	0.925–1.649
Richer	1.700**	1.202–2.318
Richest	1.445*	0.987–2.117
Age		
15–19	Ref	Ref
20–24	1.045	0.805–1.356
25–29	0.991	0.715–1.374
30–35	0.800	0.551–1.161
35–39	1.044	0.705–1.549
40–44	0.987	0.654–1.489
45–49	0.644**	0.419–0.993
Ecological zone		
Coastal zone	Ref	Ref
Forest zone	2.863***	2.393–3.426
Savannah	2.167***	1.580–2.972
Religion		
Catholic	Ref	Ref
Anglican	1.231	0.515–2.944
Methodist	1.010	0.692–1.475
Presbyterian	0.859	0.576–1.282
Pentecostal/charismatic	0.747*	0.577–0.968
Other Christian	0.571***	0.421–0.775
Islam	0.862	0.630–1.179
Traditionalist/spiritualist	0.252***	0.146–0.437
No religion	0.375***	0.237–0.595
Other	1	
Ethnicity		
Akan	Ref	Ref
Ga/dangme	0.548***	0.392–0.762
Ewe	1.110	0.880–1.454
Guan	0.651	0.401–1.057
Mole-Dagbani	1.248	0.909–1.712
Grusi	1.852	1.112–3.062
Gurma	0.470	0.319–0.692
Mande	4.382	1.783–10.733
Other	1.018	0.570–1.817
Education		
No education	Ref	Ref
Primary	1.341*	1.054–1.707
Secondary	1.999***	1.578–2.503
Higher	1.702*	1.030–2.814
Occupation		
Not Working	Ref	Ref
Working	0.977	0.802–1.191
Marital status		
Single	Ref	Ref
Married	1.396**	1.123–1.735
Cohabitation	1.012	0.792–1.296
Parity		
Zero birth	Ref	Ref
One birth	1.570**	1.190–2.073
Two births	1.840***	1.340–2.525
Three births	1.436*	1.010–2.040
Four births or more	1.652**	1.167–2.360

Source: GDHS 2014 *P < 0.05 **p < 0.01 ***p < 0.001

## Discussion

Even though the overall weighted sample registered under the scheme was slightly higher than what was reported by Kumi-Kyereme and Amo-Adjei [30] (40.2 %) it does not refute the argument made by Amu [32] that the NHIS has failed to cover 100 % of the Ghanaian population after five years as stipulated in the objectives of the scheme upon its creation in 2003. The less than 50 % NHIS ownership observed within the weighted sample may be due to the fact that people are unable to afford the cost of subscribing to the scheme as opined by Amu [32]. Boateng and Awunyor-Vitor [33] also argued that some people usually consider the premium for subscribing to the NHIS as too expensive and this serves as a barrier to their ownership of the scheme. This is particularly so for persons who pay annual premiums on the scheme; eg. Informal sector workers. The fact that ‘Richer’ and ‘Richest’ wealth quintiles were the only categories within wealth status where majority were NHIS subscribers thus underscores the role of wealth in influencing the ability of people to subscribe to health insurance as mentioned by Kumi-Kyereme and Amo-Adjei [30]. It therefore clearly justifies the inability of majority of women in the lower wealth quintiles to subscribe to the scheme.

The issue of expensiveness of yearly premium for subscribing to the NHIS was one of the reasons in 2008, prior to the general elections in Ghana, and still dominating debates in the country, a one-time premium payment on the scheme became a major issue of debate among the various contesting political parties, with the National Democratic Congress (NDC) proposing its adoption. With a one-time NHIS premium system, subscribers will be required to pay premium only once in their entire life time [34]. The major argument against this policy by opposing political parties, including the New Patriotic Party (NPP), is, however, the fact that a one-time premium would mean subscribers paying huge sums of money, which in itself, defeats the purpose of introducing such a policy in the first place; to reduce the cost of paying for the scheme by subscribers.

The findings where 40.2 % were subscribed to the scheme, may also be attributed to delays – as a result of long queues – which characterise registration/renewal of NHIS membership and utilisation of health services with insurance or dissatisfaction with quality of services received from the scheme regarding registration/renewal and health care use [10, 35]. The fact that African traditionalists were the least to own health insurance may be attributable to the fact that they largely believe in the use of herbal and/or African traditional medicine [36] at the expense of orthodox ones compared to people with other religious perspectives.

The fact that level of education predicted health insurance subscription is an indication that educational attainment cannot be ruled out when decisions regarding utilisation of health care services are concerned. It was thus obvious to note from the study that at least half of all women with secondary and higher levels of education were subscribed to the NHIS [37]. Amu [32] noted in this regard that people with high levels of education may have a higher outlook with regards to the necessity of being ready for any unforeseen health challenges and as such decide to own health insurance, as opposed to those with lower or no level of education who may not realise the level of threat that will be posed to their health and life if they are not prepared financially for any unforeseen health challenges but they eventually occur [38].

As observed in our study, a woman’s age does inform her decisions regarding health insurance subscription. Age influences perception of susceptibility to health conditions and the seriousness attached such conditions [39]. The level of susceptibility then results in decisions to either subscribe to health insurance in order to be ready for unforeseen health challenges or not to do so [38]. Women in rural areas being more likely to subscribe to health insurance than women in urban areas is an indication of the higher need for health care services documented to exist in rural areas than in urban areas [40–42]. Thus, women in the rural areas were more likely to subscribe to the health insurance so as to be able to afford health care in times of need, as they may not be able to afford health care out-of-pocket if they fall sick [43].

Our findings regarding ecological zone confirm those found by Kumi-Kyereme and Amo-Adjei [30] in which the authors posited that the highest percentage of women subscribed to the NHIS was in the forest zone while the least was from the Coastal zone. The fact that women with zero parity had the least rate of subscription to the NHIS may be because they did not require pregnancy and childbirth services which are services that make most women in their reproductive age to Ghana to subscribe to the NHIS as they are exempted from the payment of premiums [44].

## Conclusions

We found that education, wealth status, age, religion, birth parity, marriage and ecological zone predict health insurance subscription among women in their reproductive ages in Ghana. Urban dwellers, women who have never given birth, those with no or low levels of education, African traditionalists and the poor were women who largely did not subscribe to the scheme. The NHIA should therefore carry out more education than they are currently doing, in association with the National Commission for Civic Education and the Information Services Department to recruit more of these people unto the scheme. The indigent for instance should be made aware through these educational campaigns that subscription to the NHIS for them is without any charges, which will encourage them to subscribe.
